# From causes of aging to death from COVID-19

**DOI:** 10.18632/aging.103493

**Published:** 2020-06-12

**Authors:** Mikhail V. Blagosklonny

**Affiliations:** 1 Roswell Park Cancer Institute, Buffalo, NY 14263, USA

**Keywords:** aging, mTOR, rapalogs, senolytics, SARS-CoV-2, COVID-19, coronavirus

## Abstract

COVID-19 is not deadly early in life, but mortality increases exponentially with age, which is the strongest predictor of mortality. Mortality is higher in men than in women, because men age faster, and it is especially high in patients with age-related diseases, such as diabetes and hypertension, because these diseases are manifestations of aging and a measure of biological age. At its deepest level, aging (a program-like continuation of developmental growth) is driven by inappropriately high cellular functioning. The hyperfunction theory of quasi-programmed aging explains why COVID-19 vulnerability (lethality) is an age-dependent syndrome, linking it to other age-related diseases. It also explains inflammaging and immunosenescence, hyperinflammation, hyperthrombosis, and cytokine storms, all of which are associated with COVID-19 vulnerability. Anti-aging interventions, such as rapamycin, may slow aging and age-related diseases, potentially decreasing COVID-19 vulnerability.

##  

### COVID-19 vulnerability: age, diseases, gender

COVID-19 is caused by coronavirus SARS-CoV-2. Most cases of COVID-19 are asymptomatic, but some are severe and lethal. Mortality is the simplest marker of COVID-19 vulnerability. COVID-19 vulnerability can be defined as a chance of death from COVID-19, once infected.

**Age:**

In all studies conducted in all countries, the mortality rate from COVID-19 increases exponentially with age [1–11]. Exact mortality rates varied in hundreds of studies because they depend on testing and therapeutic interventions. But the rule is clear: the mortality rate is increasing exponentially with age.

**Age-related diseases:**

Mortality is especially high in patients with pre-existing conditions [6, 9, 10, 12–23].

In Italy, 99% of patients, who died, had at least one illness https://www.bloomberg.com/news/articles/2020-03-18/99-of-those-who-died-from-virus-had-other-illness-italy-says.

In other words, infected people without pre-existing diseases do not die. This may seem paradoxical because we just discussed that age is sufficient to increase mortality exponentially. This is because pre-existing conditions are manifestations of biological age, whereas aging and diseases are two sides of the same coin [24–26]. These conditions are typical age-related diseases: hypertension, diabetes, obesity, ischemic heart disease (IHD) and chronic obstructive pulmonary disease (COPD) and other diseases [9, 12–23].

Of course, not all (only some) patients with age-related diseases die from COVID-19. In other words, age-related diseases are necessary but not sufficient for mortality from COVID-19.

Age and pre-existing (age-related) diseases are interdependent. A number and severity of diseases correlate with age. An average 60 year old person has more age-related diseases than an average 50 your old person. Yet, a particular 60 year old person may have no age-related diseases, whereas a particular 50 year old person may have multiple diseases including hypertension, diabetes, obesity and cancer. In this case, it is a chronologically younger person who is biologically older. And it is the biological age that determines the likelihood of death from COVID-19.

**Male Gender:**

At the same age, the mortality rate is twice higher in men than in women [9, 27, 28], in part, because men age faster than women and, at any chronological age, men are biologically older than women [29].

So, three rules can be combined in one: COVID-19 vulnerability is determined by biological age. Biological age combines chronological age, age-related diseases and gender. A combination of all age-related diseases (and pre-diseases) is a biomarker of biological age. Figuratively, SARS-Cov-2 can “measure” biological age, which is thus the best predictor of mortality from both COVID-19 and other diseases.

### Mortality from aging compared with COVID-19 mortality

Aging can be measured as an increase in the probability of death with age. Mortality increases exponentially, starting from age 8-9. Men have a higher “normal” age-related death rate than women because men age faster than women [29].

COVID-19 mortality rate parallels the “expected” aging-related death rate (Supplementary Figure 1) and see second graph in: https://medium.com/wintoncentre/how-much-normal-risk-does-covid-represent-4539118e1196.

Chances to die from COVID-19 are proportional to chances to die from aging itself at any age. The only discrepancy between natural and COVID-19 mortality is observed below the age of 8 years old. Whereas natural death rate is relatively high, COVID-19 mortality is low (no mortality [11]). This discrepancy will be discussed later. But first how do animals, including humans, die from aging?

### Age-related diseases

Humans and other animals (including the worm [30] and the fly [31]) do not die from aging itself but from age-related diseases such as ischemic heart disease (IHD), hypertension, diabetes, cancer, Alzheimer’s and Parkinson’s diseases, age-related macular degeneration, osteoporosis and sarcopenia (As we will discuss, even seemingly non-deadly diseases such as osteoporosis can lead to deadly complications). The incidence of these diseases increases exponentially with age. Some diseases such as obesity, hypertension and diabetes develop earlier in the course of aging. Other diseases, such as Alzheimer’s disease and macular degeneration, are usually diagnosed later [32, 33]. Age-related diseases may also occur in younger people with genetic predisposition and environmental exposure hazards. But even without these factors, diseases develop because they are quasi-programmed (see “Quasi-programmed aging section”). These diseases are not diseases of civilization, as it may seem. Humans simply now live long enough to develop them. Of course, “hazards of civilization” can accelerate them at a younger age.

Aging and its diseases cannot be separated. Healthy aging, or aging without diseases, is merely a slow aging, when biological age is less than chronological age. During a period of seemingly healthy aging, pre-pre-diseases and pre-diseases are progressing until they eventually reach clinical manifestations. Thus, healthy aging progress to unhealthy and pre-diseases become diseases [34].

Age-related diseases and COVID-19 vulnerability are highly intertwined. Patients, who die from COVID-19, otherwise would die from age-related diseases such as heart disease, cancer, diabetes, hypertension, just a year later. COVID-19 approximately doubles a patient’s aging-dependent risk of dying during one year. For example, (numbers are very approximate), a sixty year old woman has 1% chance to die from aging before her 61^st^ birthday. At that age, if infected, the death rate from COVID-19 is around 1% for females. If infected, a patient has approximately doubled chances to die compared with usual age-related mortality during one year. As David Spiegelhalter put it: “getting COVID-19 is like packing a year’s worth of risk into a week or two”. https://medium.com/wintoncentre/how-much-normal-risk-does-covid-represent-4539118e1196.

Children and young adults have a very low risk of death from aging-related diseases, so that risk remains extremely low even when doubled.

Although natural mortality is relatively high in the youngest age group, especially in infants, they do not die from age-related diseases of course. Instead, infants are vulnerable to bacterial infections and candida infections due to underdeveloped immune system [35]. Low COVID-19 mortality in the pediatric age group [11] is consistent with the notion that COVID-19 vulnerability is not due to a “weak” immune system. In contrast, as we will discuss in the next section, it is hyper-functional immune response that leads to death from COVID-19 in the elderly by causing cytokine storm.

### Cytokine storm as a hyperfunction

Severe COVID-19 is characterized by hyper-inflammation, cytokine storm, acute respiratory distress syndrome (ARDS), damage to the lung, heart and kidneys [36–39].

In response to viral replication, hyperfunctional monocytes and macrophages infiltrate the lung, causing hyper-inflammation and hyper-secretion of cytokines such as interleukin (IL)-6, IL-2, IL-7, IL-1ra, interferon-γ inducible protein (IP)-10, tumor necrosis factor (TNF)-α, ferritin, monocyte chemoattractant protein (MCP)-1, macrophage inflammatory protein (MIP) 1-α, granulocyte-colony stimulating factor (G-CSF), C-reactive protein (CRP) and procalcitonin. [22, 36–42].

This leads to leukocyte recruitment, vascular permeability, edema and further pulmonary damage in vicious cycle [37, 38, 41, 43, 44]. Hyper-inflammation becomes systemic, in turn causing hyper-coagulation and thrombosis, disseminated intravascular coagulation [45]. This causes injury of distant organs such as the kidneys. Pre-existing organ damage (late stages of age-related diseases) exacerbates organ damage caused by cytokine storm [42, 43, 46]. In addition, cellular hyper-functions and systemic hyper-inflammation may lead to cellular exhaustion, such as exhaustion of lymphocytes (lymphopenia) [47–49]. Hypercoagulation is associated with hyperactive fibrinolysis and increased D-dimer blood levels [23]. Cytokine storm is a systemic hyperfunctional response (Figure 1).

**Figure 1:**
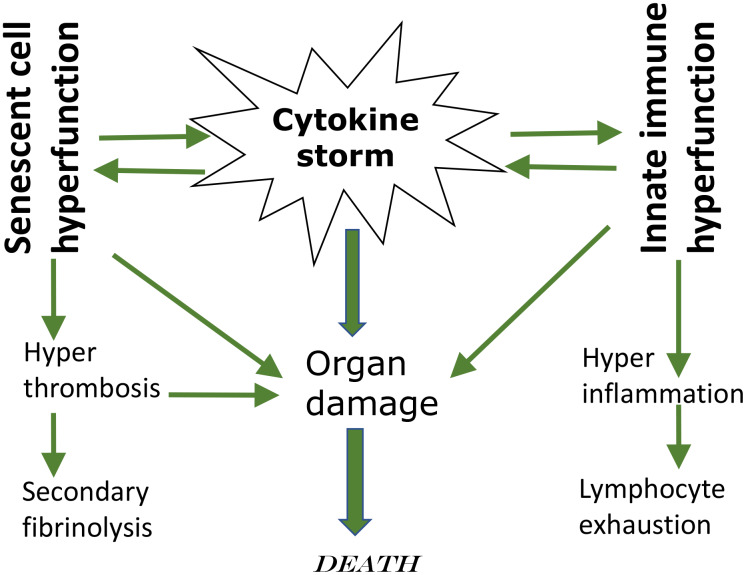
**Cytokine storm as a systemic hyperfunction.**

Of course, age-related hyperfunctional response, such as cytokine storm, is not caused by lifelong accumulation of molecular damage. Aging is not caused by molecular damage after all. Instead it’s a continuation of developmental/growth programs that lead to hyper-functions and in turn eventually to dysfunctions.

### Hyperfunction theory of quasi-programmed aging

“Quasi” means “resembling” or “seemingly, but not really.” Quasi-program of aging is not a program but a continuation of developmental programs that were not switched off upon their completion [24, 50]. They purposelessly unfold, leading to age-related diseases, secondary organ failure and death. Quasi-programmed (program-like) aging is associated with higher than optimal cellular and systemic functions, which eventually, via cellular exhaustion and organ damage, lead to functional decline (Figure 2). For example, starting from birth, blood pressure increases and continues to increase after organismal growth is completed. Therefore, hypertension is the most prevalent age-related disease. In turn, hypertension can cause organ damage: stroke, infarction and renal failure. Similarly, obesity develops in post-development as a continuation of growth (yet, it can be prevented by low caloric diets, illustrating that quasi-program of aging can be decelerated).

**Figure 2:**
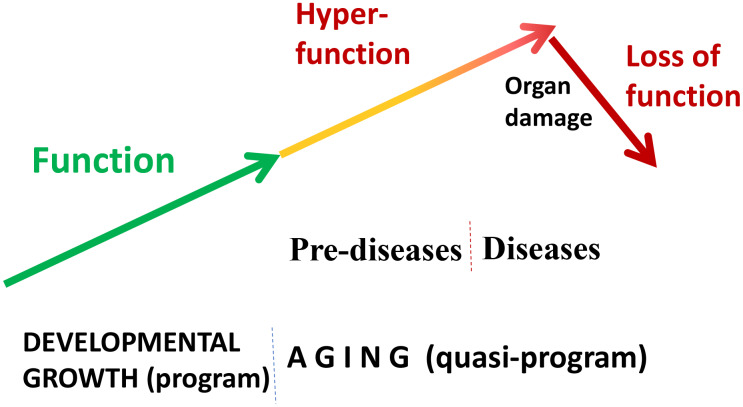
**Quasi-programmed hyperfunctional aging.** Aging is a continuation of developmental programs that were not switched off upon their completion. An increase in cellular and systemic functions (manifested as pre-diseases and then as diseases) leads to eventual organ damage and secondary loss of function.

Hyperfunction is an excessive normal cellular function: contraction by smooth muscle cells (SMC), adhesion and aggregation by blood platelets, insulin secretion by beta-cells, lipid accumulation by adipocytes, secretion by stromal and immune cells, oxidative burst by leukocytes, just to name a few. When higher than optimal, they cause vasoconstriction and hypertension, thrombosis, hyperinsulinemia, hypertrophy, hyperplasia, obesity, hyper-secretory phenotype or Senescence-associated secretory phenotype (SASP), hyper-inflammation and so on.

Hyper-function is not necessarily an absolutely increased function. It may be also insufficiently decreased function (relative hyperfunction). Levels of IGF-1 and growth hormone decrease during lifespan. Despite this decrease, IGF-1 levels are still higher than optimal (relative hyper-function) because further genetic decrease in IGF-1 levels (by genetic means) extends health span and lifespan in mammals [51–53].

Cellular hyperfunctions may eventually switch to cellular exhaustion and loss of functions at late stages. During the course of type II diabetes, mTOR overactivation and hyperinsulinemia eventually lead to beta-cell exhaustion and insulin insufficiency, from pre-diabetes to diabetes [54, 55]. As another example, after puberty, hyperstimulation of the ovary eventually leads to oocyte exhaustion and menopause (see Figure 3 in ref. [29]). Depletion of naïve lymphocytes is another example, as reviewed here later. Age-related alterations are mostly noticed when they switch to functional decline, which is a late event.

**Figure 3:**
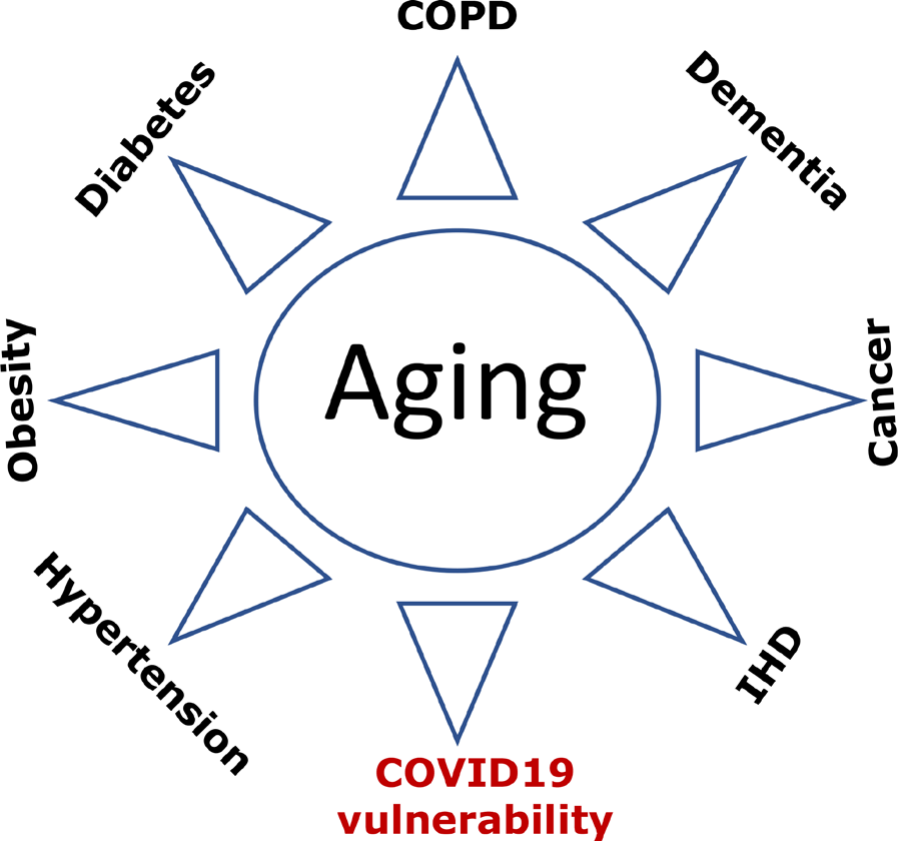
**COVID-19 vulnerability as an age-related disease.** Age-related diseases, including COVID-19 vulnerability, are manifestations of aging. Abbreviations: Ischemic heart disease (IHD); Chronic obstructive pulmonary disease (COPD).

In some cases, functional decline can be primary and programmed. For example, thymus involution (replacement of T cells by adipocytes) starts early in life, accelerates at puberty and continues later. Still loss of thymocytes and their niches may be in part due to adipocyte hyperplasia and hypertrophy [56]. In fact, obesity accelerates involution, whereas calorie restriction decelerates it [57, 58]. Furthermore, the oblation of sex hormones decelerates or even reverses thymus involution [59]. Thus, involution is triggered by adipocyte hyperplasia and increased production of sex hormones during puberty [56].

Quasi-programmed aging is not driven by molecular damage. It is driven by nutrient/hormone/cytokine-sensing and growth-promoting signaling pathways such as Target of Rapamycin (TOR; mTOR), which are involved in developmental growth and later cause hyperfunctional aging and its diseases [24, 26].

### Covid-19 vulnerability as an age-related syndrome

What is the cause-effect relationship between age-related diseases and COVID-19 lethality? Do patients die from age-related diseases, complicated by COVID-19? Or, in contrast, do these various diseases make COVID-19 infection lethal? Both scenarios take place to some extent. However, the relationship is mostly indirect. Both age-related diseases and COVID-vulnerability result from the same underlying cause (Figure 3). This is why they are highly correlated. The cause is aging itself. Aging is manifested by a sum of deadly - and not so deadly - diseases and conditions ranging from cancer to grey hair. Although not all diseases seem to be deadly, they can cause complications such as stroke, ventricular fibrillation, renal failure, lung edema. Even sarcopenia and osteoporosis lead to falls and broken bones culminating in a deadly sequence of events. Cosmetic manifestations such as aging spots and wrinkles, while not deadly by themselves, can be manifestations of other diseases. For example, baldness correlates with prostate enlargement [60], and the later can lead to urinary obstruction and renal failure.

Diseases occur together. For example, chronic obstructive pulmonary disease (COPD) is associated with diabetes, cardiovascular disease and hypertension [61]. If a person has one disease (e.g., diabetes), this patient has higher chances of having other diseases (e.g., hypertension, IHD, cancer) or conditions, including COVID-19 vulnerability, which is revealed only during infection but can be predicted by pre-existing diseases.

Aging is initially driven by an increase in cellular and systemic functions (hyperfunction), leading to age-related conditions. For example, hypertension is a systemic hyperfunction due to hyperfunction of multiple cell types such as arterial smooth muscle cells (aSMC). Similarly, COVID-19-vulnerability is associated with hyperfunction of inflammatory cells that, in response to COVID-19 infection, causes cytokine storm, hyper-coagulation and damage of the lung and distant organs.

The COVID-19 vulnerability syndrome is an aging-related disease, strictly dependent on biological age, associated with other age-related diseases, and exemplified by hyper-functional response to infection.

### Inflamm-aging and immunosenescence

With hundreds of cell types acting in concert, the immune system is so complex that we cannot discuss age-related alterations without oversimplification. The most noticeable alteration is that memory T and B cells replace naive T and B cells [62]. (This seems natural since life-long exposure to pathogens replaces naïve cells by memory cells). Replacement of naïve immune cells decreases adaptive responses to novel antigens such as SARS-CoV-2. In contrast, immune protection by memory T cells from viral re-infection with known pathogens is usually increased with age [62].

Immune responses are roughly divided into (a) innate responses, carried mostly by neutrophils, macrophages and NK cells, which react to pathogen rapidly and nonspecifically, and (b) adaptive responses, carried by T and B lymphocytes, which are delayed, slower and specific (e.g., antigen-specific clonal expansion of T and B lymphocytes and antibody production by B lymphocytes) [63–65]. In the elderly, immune responses to SARS-CoV-1/2 are “stuck in innate immunity,” with insufficient progression to adaptive immunity [37]. However, decline in adaptive response, such as antibody production, plays little role in COVID-19 mortality. It is hyper-functional innate immunity, hyper-inflammation, cytokine storm and hyper-coagulation that lead to organ failure and death. In agreement, hyper inflammatory response rather than high virus numbers leads to death of SARS-CoV-infected old nonhuman primates [66].

Aging is associated with diseases of immune hyper-function such as autoimmune disorders with paradoxical increase in certain signaling pathways and cytokine levels [67–69].

In the elderly, innate immune cells are in a state of sustained activation, producing pro-inflammatory cytokines [67, 70–72]. Increased pro-inflammatory activity by the innate immune system, especially by monocytes/macrophages, is a state of alertness and hyper-reactivity on the cost of potential age-related inflammatory diseases [67, 70–72]. Whereas some functions are decreased, others are increased. According to the inflamm-aging concept, innate immune system overtakes adaptive immune system in aging. Cause-effect relationships are bi-directional: immunosenescence (namely, a decrease in adaptive response) is a cause and consequence of inflamm-aging [67, 70–72].

We can consider inflamm-aging as an example of hyper-function. While some functions are decreased, others are increased. Hyper-function is damaging. (In analogy, increased electric power, without an adaptor, would damage a laptop). Damaging hyper-functions can lead to loss of function and cellular exhaustion. And vice versa, loss of function may cause compensatory hyper-functions of another components.

### Cellular senescence as a continuation of growth

Cellular senescence is a continuation of cellular growth, when actual growth is completed [73, 74]. In proliferating cells, cellular mass growth is balanced by cell division. Cells grow in size and then divide. When the cell cycle is blocked (e.g., p21 and p16), then growth-promoting pathways such as mTOR and MAPK drive conversion to senescence (geroconversion) [24, 74, 75]. During geroconversion, cells become hypertrophic and “fat”. Cellular functions increase: hyper-secretion and lysosomal hyper-function are manifested by SASP and beta-Gal staining. Hyper-activated growth-promoting pathways cause compensatory resistance to growth factors/insulin, permanent loss of re-proliferative potential [74]. Rapamycin, everolimus, pan-mTOR and MAPK inhibitors slows down geroconversion, maintaining reversible quiescence instead of senescence [73, 76–88].

Geroconversion is a continuation of cellular growth [73, 74]. Similarly, aging is a continuation of developmental growth (see Figure 1 in ref. [89]). When the developmental program is completed, it becomes a quasi-program of aging. As discussed in detail, chronically activated nutrient-sensing and growth-promoting pathways drive age-related diseases, culminating in organismal death [24, 26].

Age-related diseases are quasi-programmed. Aging is a common cause of age-related diseases, a sum of all age-related diseases. They are diseases of hyper-function, secondary hypo-function and compensation reactions [25]; they are deadly manifestations of aging.

From activation of cellular functions to systemic hyperfunctions, from diseases to organ damage and death, hyperfunction theory of quasi-programmed aging describes the sequence of events [26]. And as discussed in 2006, suppression of aging by gero-suppressants, such as rapamycin, will prevent and treat all age-related diseases [24]. This point of view is becoming widely accepted and, in recent literature, quasi-programmed model of diseases (2006) is called “geroscience hypothesis” [2, 90].

### Figuratively, rapamycin rejuvenates immunity [91].

If aging were functional decline due to accumulation of molecular damage, then it would be near to impossible to restore functions and rejuvenate the immune system. In contrast, if functional decline is secondary to hyperfunctions (see Figure 2 in ref. [89]), these hyperfunctions can be suppressed pharmacologically to restore lost functions. Typical drugs are inhibitors of their targets, rather than activators, so they decrease functions of their targets. By decreasing hyper-functions, which otherwise lead to secondary loss of functions, rapamycin may restore “lost” functions (Figure 4).

**Figure 4:**
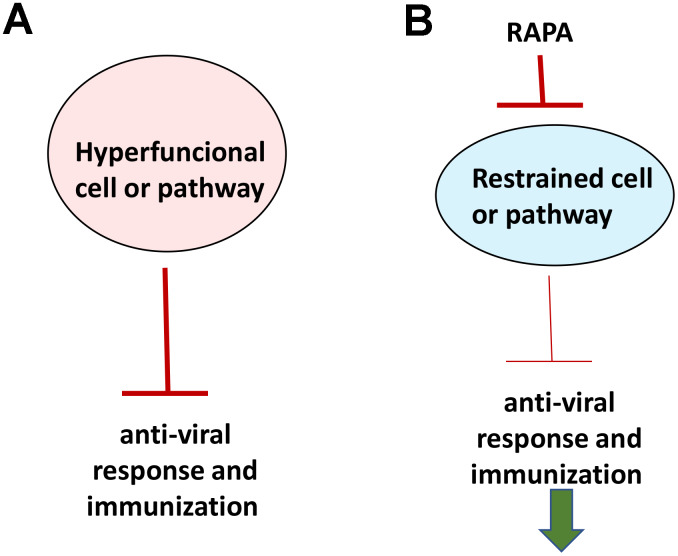
**Rejuvenating immunity by inhibiting hyperfunction.** (**A**) Specific hyper-functional cells (or signaling pathways) can inhibit some other cell types (or pathways) that are needed for proper anti-viral response and immunization. (**B**) By inhibiting hyper-functional cells or pathways, rapamycin can reactivate “loss-of-function” otherwise suppressed by hyper-functional cells or pathways.

Rapamycin improves vaccination against viruses such as influenza in old mice, monkeys and humans [92–100]. Importantly, rapamycin increases pathogen-specific but not graft-reactive CD8+ T cell responses [95, 101]. Therefore, rapamycin and everolimus can both be used to prevent donor organ rejection and improve adaptive immunity against new pathogens [96].

Differentiation is an increase of tissue-specific cellular functions. Terminally differentiated B, T, and NK cells can rapidly react to already known pathogens [102]. Decrease in naïve T and B lymphocytes (and thus diminished response to novel antigens) results in part from cellular hyper-differentiation in the immune system [64, 103]. Hyper-functional differentiation can be counteracted by rapamycin [98].

As another example, age-related exhaustion of stem cells is partially due to loss of quiescence caused by growth over-stimulation [92, 104–106]**.** In general, senescent cells characterized by hyper-proliferative drive coupled with cell cycle arrest [77]. In young mice, mTOR hyper-activation causes senescence of hematopoietic stem cells (HSC) and decreases lymphopoiesis [92]. In old mice, rapamycin rejuvenates hematopoiesis, and improves vaccination against influenza virus [92].

Third, production of lymphoid cells may be decreased because of disruption of hypoxic niches due to adipocytes hyperplasia [56, 107]. Hypoxic niches can preserve HSC [108, 109] probably because hypoxia inhibits mTOR and cellular senescence [110]. In agreement, rapamycin preserves HSCs [92, 98, 111, 112] reduces the proportion of memory cells and maintains a pool of naïve T cells [92, 98].

Fourth, growth factor (GF)- and insulin-resistance is loss of function because cells cannot respond to GF/insulin. But it may be caused by over-activated mTOR, which via S6K/IRS feedback loop blocks insulin and GF signaling. Rapamycin abrogates the loop restoring signaling [113–118].

### Anti-aging medicine

A high prevalence of age-related diseases, often called “diseases of civilization,” is a success story of modern medicine. In the past, most people did not live long enough to develop age-related diseases and those who developed them died soon after. Due to medical advances, people survive to 85 on average, despite suffering from age-related diseases. Standard medicine preferentially extends life span, without necessarily affecting health span (see Figure 3 in ref. [119]). For example, defibrillation and coronary stenting can save life but not cure heart disease. It is anti-aging interventions that extend health span, delaying diseases, thus extending lifespan. Aging is a common cause of all age-related diseases. By suppressing aging, anti-aging interventions may delay all age-related diseases [119].

As a well-known example, low calorie diets such as calorie restriction, intermittent fasting, and low carbohydrate diets extend both health and lifespan. Figuratively, low calorie diets prolong life by improving health. Nutrients and obesity activate growth-promoting pathways (e.g., mTOR), thus accelerating development of quasi-programmed (age-related) diseases. Obesity is associated with all age-related diseases from cancer to Alzheimer’s and from diabetes to sarcopenia. COVID-19 vulnerability is also associated with obesity [9, 19, 20, 22]. According to hyperfunction theory, obesity accelerates aging and all age-related conditions including COVID-19 vulnerability.

Diabetes is one of main risk factors of death in COVID-19 [5, 6, 12, 13, 15, 21]. Can type 2 diabetes, an age-related disease, be reversed? In remarkable studies, it was shown that a brief course (6-8 weeks) of very low calorie diets (VLCDs) can reverse type II diabetes. In one study, VLCD reversed diabetes in 46% of patients with up to a 6-year history of diabetes [120]. VLCD is most effective for its prevention and at early stages of diabetes [121]. This anti-aging modality is so simple that remission can be achieved at home by health-motivated individuals [122]. Simultaneously, it treats other age-related diseases such hypertension [123]. Obesity is associated with other diseases of hyperfunction from diabetes and sarcopenia to cancer and Alzheimer’s’ disease. Since age-related diseases are predictors of COVID-19 mortality, VLCD in theory may decrease COVID-19 vulnerability.

### Rapamycin and everolimus as anti-aging drugs

In the soil of Easter Island, a complex bacteria produces anti-fungal antibiotic rapamycin to suppress yeast growth but, as a by-product, it also suppresses yeast aging (quasi-programed aging is a continuation of growth). Approved for human use in 1999, Rapamycin (Sirolimus) and its close analog Everolimus are widely used in several diseases including cancer and organ transplantation. Hundreds of clinical trials (and twenty years of clinical practice) have ensured their safety and good tolerability especially in healthy older adults [119].

Currently, several anti-aging clinics prescribe rapamycin out of label to prevent age-related diseases and slow aging. Hundreds of recent reviews discussed rapamycin and everolimus in detail, so I will just emphasize a few points:

1. Crucial prediction of hyper-function theory of quasi-programmed aging in 2006 was that rapamycin will slow aging, extend healthspan and lifespan and decrease all age-related [124]. It has been confirmed: it extends lifespan in animals from worm to mammals. In some strains of short-lived mutant mice, it extends life span two fold [98, 125].
2. Rapamycin slows geroconversion to cellular senescence in cell culture [74].
3. mTOR is a potential therapeutic target in chronic obstructive pulmonary disease COPD [126], [127]. Rapamycin (sirolimus) is already approved and successfully used in lymphangioleiomyomatosis (LAM), a progressive, cystic lung disease, associated with inappropriate activation of mTOR [128]. Long-term daily use of rapamycin improves lung function without causing serious side effects (and of course no even minor side effects in the lung, given that rapamycin improves lung function) [128].
4. Despite widespread misunderstanding, rapamycin and everolimus do not cause diabetes. In contrast, they prevent diabetic complications in animals with diabetes (see for references [129]). In rodents, in some conditions they may cause symptoms of starvation pseudo-diabetes similar to prolong fasting and ketogenic diet [129]. Although, the *Johnson* study found a slight but significant correlation between Medicare billing for insulin and the use of rapamycin in renal transplant patients, this correlation was mechanistically explained by interaction of rapamycin with two other drugs used in the same patients [130, 131]. In cancer patients, everolimus may cause reversible hyperglycemia as a mild, infrequent and reversible side effect after several weeks of daily high doses of everolimus and rapamycin [132]. Mechanistically, everolimus decrease insulin production, not causing insulin resistance [132]. If anything, everolimus and rapamycin can be considered to treat complications of type II diabetes and prevent hyperinsulinemia and obesity ([129] and references within). What actually contributes to type 2 diabetes is excess of nutrients (and especially carbohydrates), which activate mTOR and cause hyperinsulinemia and insulin resistance.

### Potential applications of rapamycin/everolimus to COVID-19

As soon as COVID-19 epidemic started, it become clear that COVID-19 vulnerability is an aging-dependent condition and the use of rapamycin (Sirolimus) was immediately suggested by independent researchers [1, 3, 133–137]. These proposals were based on a mixture of several rationales, which need to be clearly distinguished. In theory, there are at least three independent applications of rapamycin and everolimus for COVID-19. Currently, they all are still hypothetical.

1. Anti-aging effect (Figure 5). By decreasing biological age and preventing age-related diseases, a long-term rapamycin therapy may in theory decrease COVID-19 mortality rate in the elderly. Anti-aging application is especially important because it is beneficial regardless of COVID-19. After all, mortality rate from aging and its diseases is 100%, causing more than 2 million deaths in the USA annually. Continuous use of rapamycin is expected to improve health, decrease age-related diseases and extend healthy lifespan, rendering individuals less vulnerable, when infected with the virus.
2. Rejuvenating immunity. As we discussed in section “Figuratively, rapamycin rejuvenates immunity” [91], mTOR inhibitors can improve immunity to viral infections, improve immunization and vaccination to some viruses such as flu [92–100, 111, 112, 138]. In addition, viruses such as flu [139] and coronavirus (MERS-CoV) [140] depend on mTOR activity for replication. Currently, however, there are no data regarding COVID-19. Although aimed to evaluate safety, Phase 1 clinical trial “Sirolimus in COVID-19 Phase 1 (SirCO-1)” may reveal anti-viral effects too https://clinicaltrials.gov/ct2/show/NCT04371640.

**Figure 5:**
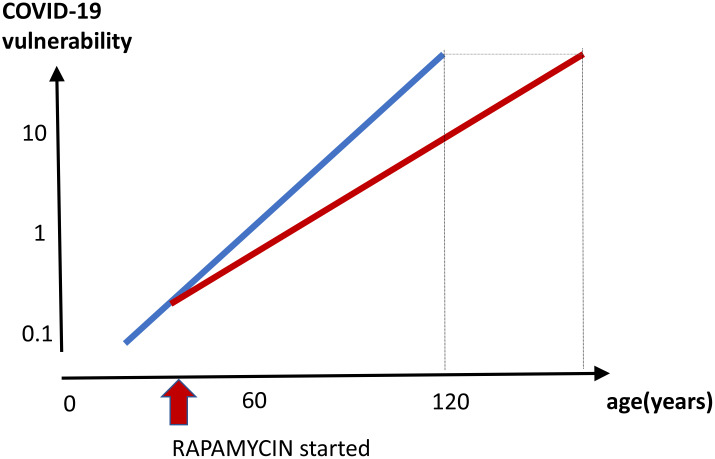
**Prevention of COVID-19 vulnerability by staying young.** Hypothetical graph in the absence of COVID-19. COVID-19 vulnerability (log scale) increases exponentially with age (blue line). The line ends at age 120, a maximum recorded age for humans. In theory, a continuous rapamycin treatment would slow down an increase of the vulnerability with age (red line). The increase is still logarithmic but at a different slope, because rapamycin slows the aging process. The maximum lifespan, in the absence of COVID-19, is extended because the 100% natural death threshold is achieved later.

3. Potential suppression of cytokine storm and hyper-inflammation (Figure 1). As we discussed in the section “Cytokine storm is a hyperfunction”, cytokine storm and hyper-inflammation is a main cause of death in COVID-19 pneumonia [36–40, 42, 45, 135, 141–143] Rapamycin, an anti-inflammatory agent, inhibits hyper-functions, cellular senescence and decrease secretion of cytokines ([74, 81, 144]. Rapamycin inhibits the Jak2/Stat4 signaling pathway [145] and reduces IF-γ and TNF-α levels [112]. Rapamycin (Sirolimus) treatment improves outcomes in patients with severe H1N1 pneumonia and acute respiratory failure and was associated with improvement in virus clearance, and shortened ventilator days [146]. Clinical trial “Sirolimus Treatment in Hospitalized Patients With COVID-19 Pneumonia (SCOPE)” has been started https://clinicaltrials.gov/ct2/show/NCT04341675.

### Disclaimer

This review is intended for a professional audience, to stimulate new ideas and to aid the global efforts to develop effective treatments for COVID-19 disease. This article does not represent medical advice or recommendations to patients. The media should exercise caution and seek expert medical advice for interpretation, when referring to this article.

## Supplementary Material

Supplementary Figure 1
