# Supplementary material for: From causes of aging to death from COVID-19
Source: Aging (Albany NY). 2020 Jun 12;12(11):10004–21. doi: 10.18632/aging.103493 (PMC7346074; doi:10.18632/aging.103493)
Supplement: Supplementary Figure 1 [file aging-12-103493-s001..pdf]

SUPPLEMENTARY FIGURE

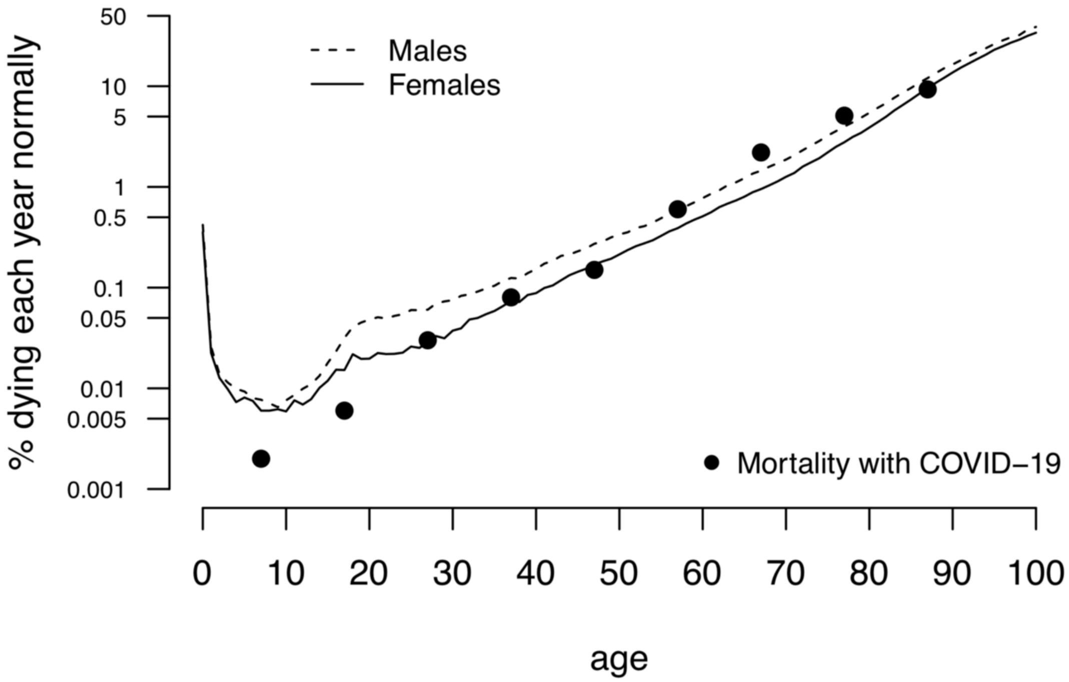

Supplementary Figure 1. The mortality risk with COVID-19 superimposed on background annual risk. Annual risk of death (hazard) for England and Wales, 2016–2018, from Office for National Statistics. https://medium.com/wintoncentre/how-much-normal-risk-does-covid-represent-4539118e1196.
